# Supplementary material for: Tumour acidosis remodels the glycocalyx to control lipid scavenging and ferroptosis
Source: Nat Cell Biol. 2026 Feb 11;28(3):567–80. doi: 10.1038/s41556-026-01879-y (PMC12992114; doi:10.1038/s41556-026-01879-y)
Supplement: Supplementary file 1 — Reporting Summary [file 41556_2026_1879_MOESM1_ESM.pdf]

nature portfolio

Corresponding author(s): Mattias Belting

Last updated by author(s): Jan 5, 2026

Reporting Summary

Nature Portfolio wishes to improve the reproducibility of the work that we publish. This form provides structure for consistency and transparency in reporting. For further information on Nature Portfolio policies, see our Editorial Policies and the Editorial Policy Checklist.

Statistics

For all statistical analyses, confirm that the following items are present in the figure legend, table legend, main text, or Methods section.

n/a	Confirmed	
☐	☒	The exact sample size (*n*) for each experimental group/condition, given as a discrete number and unit of measurement
☐	☒	A statement on whether measurements were taken from distinct samples or whether the same sample was measured repeatedly
☐	☒	The statistical test(s) used AND whether they are one- or two-sided<br>*Only common tests should be described solely by name; describe more complex techniques in the Methods section.*
☐	☒	A description of all covariates tested
☐	☒	A description of any assumptions or corrections, such as tests of normality and adjustment for multiple comparisons
☐	☒	A full description of the statistical parameters including central tendency (e.g. means) or other basic estimates (e.g. regression coefficient) AND variation (e.g. standard deviation) or associated estimates of uncertainty (e.g. confidence intervals)
☐	☒	For null hypothesis testing, the test statistic (e.g. *F*, *t*, *r*) with confidence intervals, effect sizes, degrees of freedom and *P* value noted<br>*Give P values as exact values whenever suitable.*
☒	☐	For Bayesian analysis, information on the choice of priors and Markov chain Monte Carlo settings
☒	☐	For hierarchical and complex designs, identification of the appropriate level for tests and full reporting of outcomes
☐	☒	Estimates of effect sizes (e.g. Cohen's *d*, Pearson's *r*), indicating how they were calculated

Our web collection on statistics for biologists contains articles on many of the points above.

Software and code

Policy information about availability of computer code

Data collection

Four imaging platforms were used: LSM710 Airyscan confocal platform (Carl Zeiss AG, Oberkochen, Germany), operating under ZEN 2.1 (black); LSM980 confocal platform (Zeiss), operating under ZEN 3.8.2 (blue); Axio Scan.Z1 slide scanner (Zeiss), operating under ZEN 3.1 (blue); and Incucyte® S3 Live-Cell Analysis System (Sartorius), operating under the 2024B controller version (Sartorius).
Flow cytometry analyses were performed on Accuri C6 Flow Cytometer (BD Biosciences).
PCR was performed on StepOnePlus Real-Time PCR System (Applied Biosystems).
Extracellular vesicles (EVs) were characterized using the Exoid platform (Izon Science Ltd).
Laser Capture Microdissection (LCM) was performed on the Zeiss PALM system (Zeiss).
For CUT&RUN analyses, libraries were sequenced as PE150 on a NovaSeqX Sequencing System (Illumina).
Gene expression profiling was performed using either the Affymetrix Clariom D Pico Gene Array or the Illumina Human HT-12 v4 Expression BeadChip.

Data analysis

Confocal/scanner images were processed and quantified using the following softwares: ZEN 3.1 (blue), ImageJ software (v1.54p), CellProfiler (v4.2.1) and MATLAB (v2018a).
Incucyte S3 integrated software (v2022B Rev2 or v2024B) was used for analysis and visualization of Incucyte images.
Flow cytometry data were analyzed using BD CSampler™ Plus Software v1.0.27.1 (BD Biosciences) and FlowJo (v10).
Western blot images were processed by ImageJ software (v1.54p) or Image Studio Lite (v5.3.5).
For CUT&RUN analyses: Data were processed using FastQC (https://www.bioinformatics.babraham.ac.uk/projects/fastqc/), Trimmomatic (v0.39), Bowtie2 (v2.4.5), Subread (v2.1.1), SEACR (v1.3), and R (v4.x) with Bioconductor (v3.20). Gene annotation was performed using EnsDb.Hsapiens.v86. Data visualization was carried out using Galaxy (usegalaxy.org; v25.0.rc1) and the UCSC Genome Browser (hg38).

For gene expression analysis: Gene array analyses were performed using R (v4.4.2) with RStudio, and the oligo (v1.70.0), affycoretools (v1.78.0), clariomdhumantranscriptcluster.db (v8.8.0), illuminaHumanv4.db (v1.26.0), and org.Hs.eg.db (v3.20.0) annotation packages.
Differential gene expression analysis was performed using limma (v3.62.1) and the stats package (v4.4.2).
Gene set enrichment analysis (GSEA) was conducted using clusterProfiler (v4.14.4), msigdbr (v7.5.1), and igraph (v2.1.2).
Signature scoring was performed using hacksig (v0.1.2) and ggExtra (v0.10.0).
Gene expression data were visualized using ggplot2 (v3.5.1).
All R code and processed data scripts for mRNA array data are available from Zenodo at https://doi.org/10.5281/zenodo.17581666, which provides the full reproducible analysis pipeline.
R (v4.4.2) with RStudio using clusterProfiler (v4.14.4) and GraphPad Prism (v10.5.0) were used to create figures and perform statistical testing.
Schematics were created with BioRender.com.
Figure composition was performed with Adobe Illustrator v.28.6.

For manuscripts utilizing custom algorithms or software that are central to the research but not yet described in published literature, software must be made available to editors and reviewers. We strongly encourage code deposition in a community repository (e.g. GitHub). See the Nature Portfolio guidelines for submitting code & software for further information.

Data

Policy information about availability of data

All manuscripts must include a data availability statement. This statement should provide the following information, where applicable:

- Accession codes, unique identifiers, or web links for publicly available datasets
- A description of any restrictions on data availability
- For clinical datasets or third party data, please ensure that the statement adheres to our policy

All data supporting the graphs in this paper, as well as all unprocessed blot images, are available in the Source Data files. Source data is available for Figs. 2c, f-h, 3b, d, g, i-k, 4a, b, d-h, j, k, 5a-c, e, g-j, 6a-e, g, h, j, 7a-f, 8a-c, e-g and Extended Data Figs. 1e, h, 3a, d-g, i, j, 4b-d, f, h, 5b-e, g-m, 6a-c, e-h, j-p, 7a, f-i, 8a-f, h, j-l, n, 9b-j, 10a-g. Additional quality control metrics and information for CUT & RUN analyses are provided in Supplementary Table 1. The mRNA array datasets generated have been deposited in the NCBI Gene Expression Omnibus (GEO) under accession codes GES300758, GSE300765, GSE300768 and GSE300771. The CUT & RUN datasets are available in GEO under accession code GSE300142. Imaging files and all other raw data files are available from the corresponding author (due to the size of this material).

Research involving human participants, their data, or biological material

Policy information about studies with human participants or human data. See also policy information about sex, gender (identity/presentation), and sexual orientation and race, ethnicity and racism.

Reporting on sex and gender	No exclusion criteria related to sex and gender were present for the study.
Reporting on race, ethnicity, or other socially relevant groupings	Not applicable. The experiments in this study did not involve reporting on race, ethnicity, or other socially relevant groupings. All human participant data used were analyzed in a manner that did not require stratification by these characteristics.
Population characteristics	Patients with glioma (WHO grade 2 to 4) or CNS metastasis (from kidney cancer, malignant melanoma or lung cancer) were collected from both male and female participants, age ≥ 18 years.
Recruitment	Clinical samples were collected from patients referred to the Neurosurgery Department at Lund University Hospital. Inclusion criteria were age 18 y or above, WHO performance status 0 to 4, and ability to give written informed consent. There was no self-selection or other bias in recruitment. Participation was voluntary, and no financial or other incentives were provided. Patients were diagnosed by routine MRI of the brain and surgical and pathological procedures, received standard oncological treatment, and were followed up according to local and national recommendations.
Ethics oversight	The study was carried out according to the ICH/GCP guidelines, in agreement with the Helsinki declaration, and approved by the local ethics committee, Lund University (Dnr. 454 2018/37).

Note that full information on the approval of the study protocol must also be provided in the manuscript.

Field-specific reporting

Please select the one below that is the best fit for your research. If you are not sure, read the appropriate sections before making your selection.

☒ Life sciences ☐ Behavioural & social sciences ☐ Ecological, evolutionary & environmental sciences

For a reference copy of the document with all sections, see nature.com/documents/nr-reporting-summary-flat.pdf

Life sciences study design

All studies must disclose on these points even when the disclosure is negative.

Sample size	No statistical methods were used to pre-determine sample sizes, but our sample sizes are similar to those reported in previous publications,

Sample size	as referenced in "Statistics and reproducibility" in the Method section.<br>Sample size and independent biological replicates are detailed in figure legends. A minimum of n = 2 replicates was applied for all assays. Briefly, all in vitro assays included a minimum of n = 3 biological replicates, defined as independent culture of cells, to ensure statistical robustness. For flow cytometry, at least 10,000 cells were analyzed per biological replicate.<br>Patient-derived sample sizes were determined based on feasibility and previous experience, with 2 to 10 independent samples used per experiment, depending on the assay. Key experiments were conducted using tissue from at least 10 glioblastoma (GBM) patients and RNA analysis was performed on pooled tissue from 5 GBM patients.
Data exclusions	No animals or other data points were excluded from the analyses.
Replication	All experiments involving in vitro cell culture were reliably reproduced and validated with at least three biological replicates in a minimum of two independent experiments, unless otherwise indicated in the figure legends. All replications were successful. When feasible, each independent experiment also included technical replicates. All results are presented as the mean ± either the standard error of the mean (SEM) or standard deviation (SD), as specified. For in vivo analyses, a minimum of n = 6 mice per group was used.
Randomization	For in vitro studies, randomization was not applicable; however, all cell lines/organoids were treated identically without prior designation. For in vivo mouse experiments involving drug treatment, same aged female mice were randomly assigned into experimental groups.
Blinding	Data collection and analysis were not performed blind to the conditions of the experiments.<br>However, data in all experiments were quantified in automated ways using various software. All processing conditions were applied uniformly across sample sets to avoid bias. In vitro treatment studies were analyzed and quantified in automated ways using Live-Cell Analysis System and Incucyte® S3 software. Microscopic images were captured randomly and analyzed in a blinded manner. In vivo data collection were not performed blinded as all cages were required to clearly label mouse treatment details.

Reporting for specific materials, systems and methods

We require information from authors about some types of materials, experimental systems and methods used in many studies. Here, indicate whether each material, system or method listed is relevant to your study. If you are not sure if a list item applies to your research, read the appropriate section before selecting a response.

Materials & experimental systems

n/a	Involved in the study	
☐	☒	Antibodies
☐	☒	Eukaryotic cell lines
☒	☐	Palaeontology and archaeology
☐	☒	Animals and other organisms
☒	☐	Clinical data
☒	☐	Dual use research of concern
☒	☐	Plants

Methods

n/a	Involved in the study	
☐	☒	ChIP-seq
☐	☒	Flow cytometry
☐	☒	MRI-based neuroimaging

Antibodies

Antibodies used	The following antibodies were used for: α-Tubulin (clone DM1A, ab7291, WB: 1:10,000), CD63 (clone MEM-259, ab8219, WB: 1:1,000), Syndecan-1 (clone EPR6454, ab128936, IF/Flow Cyt: 1:500, WB: 1:3,000), EEA1 (ab2900, WB: 1:1,000), Flotillin1 (ab41927, WB: 1:1,000), TSG101 (ab30871, WB: 1:1,000), β-actin (ab8227, WB: 1:10,000), CD9 (clone EPR2949, ab92726, WB: 1:1,000), GPX4 (clone EPNCIR144, ab125066, WB: 1:1,000); all from Abcam. Mouse CD31 (clone MEC 13.3, 553371 IF 1:100) from BD Biosciences. CA9 (clone M75, AB1001, IF: 1:200) from Bioscience Slovakia. CD68 (clone D4B9C, 76437, IF 1:800), HIF-2α (clone D6T8V, 59973, WB: 1:1,000), SNAIL (clone C15D3, 3879, WB: 1:2,000), total-SMAD2 (clone D43B4, 5339, WB: 1:2,000), Phospho-SMAD2 (Ser465/467) (clone 138D4, 3108, WB:1:2,000), TGF-β (3711, WB: 1:2,000); all from Cell Signaling. Human CD31 (Clone JC70A, M0823, IF: 1:50) from Dako. HIF-1α (GTX127309, WB: 1:1,000) from GeneTex. Malondialdehyde (clone 6H6, MA5-27559, IF: 1:50), SLC7A11 (clone A7C6-R, MA5-44922, IF: 1:200); both from Invitrogen. Chondroitinase ABC (ChABC) (clone 1E10, NBP1-96141, IF:100), apoE (clone WUE-4, NB110-60531, WB: 1:500); both from Novus Biologicals. CS (clone CS-56, C8035, IF/Flow Cyt: 1:200) from Sigma-Aldrich. Single chain fragment variable (scFv) HS (clone, AO4B08, IF/Flow Cyt: 1:50), CS (clone GD3G7, IF/Flow Cyt: 1:50), CS (clone IO3H10, IF/Flow Cyt: 1:50) (kindly provided by Dr. Toin H. van Kuppevelt) and used together with mouse anti-VSV (clone P5D4, V5507, IF/Flow Cyt: 1:500) or rabbit anti-VSV (V4888, IF/Flow Cyt: 1:500); all from Sigma-Aldrich.<br>The following secondary antibodies were used: Horseradish-peroxidase-conjugated anti-rabbit (7074, WB 1:10,000) from Cell Signaling or anti-mouse (a9044, WB: 1:10,000) from Sigma-Aldrich. Goat anti-mouse Alexa Fluor 488 (A1100, 1:500), Alexa Fluor 546 (A11030, 1:500), Alexa Fluor 647 (A21235, 1:500) or Goat anti-rabbit Alexa Fluor 488 (A11008, 1:500), Alexa Fluor 546 (A11010, 1:500), Alexa Fluor 647 (A21244, 1:500); Streptavidin Alexa Fluor 488 (S32354, 1:500), Streptavidin Alexa Fluor 546 (S11225, 1:500) or Streptavidin Alexa Fluor 647 (S21374, 1:500); all from Invitrogen.
Validation	All commercial antibodies have been validated by the manufacturer and used according to the applications recommended by their respective manufacturers.<br>Single chain fragment variable (scFv) anti-CS clone GD3G7, clone IO3H10 and anti-HS clone AO4B08, were validated in previous

studies as indicated by the respective references under the Methods section "Compounds and antibodies".

Eukaryotic cell lines

Policy information about cell lines and Sex and Gender in Research

Cell line source(s)	Human Glioblastoma (GBM) cell line: U87MG was purchased from the American Type Culture Collection (ATCC, Cat# HTB-14). Human pancreatic cancer cell line: PANC-1 was purchased from ATCC (Cat# CRL-1469).<br>Human GBM primary cell cultures U3054MG, U3047MG, U3017MG were provided by HGCC, Uppsala.
Authentication	None of these cell lines were authentication in this study.
Mycoplasma contamination	All cell lines used in this study tested negative for mycoplasma contamination.
Commonly misidentified lines<br>(See ICLAC register)	No commonly misidentified cell lines were used.

Animals and other research organisms

Policy information about studies involving animals; ARRIVE guidelines recommended for reporting animal research, and Sex and Gender in Research

Laboratory animals	For all in vivo experiments of the study female NOD SCID gamma (NSG) mice, aged 5-7 weeks, obtained from the Jackson Laboratory (JAX), were used. GBM models included: (1) patient-derived xenograft (PDX) model using U3054MG cells, and (2) a cell line derived xenograft model using U87MG pH 7.4/NA or pH 6.4/AA cells.<br>All mice were housed in a specific pathogen-free facility with standard access to water and laboratory diet. Animals were group-housed under a 12-hour light/dark cycle, with an ambient temperature of 68–79°F and relative humidity of 30–70%.<br>Mice were monitored daily, and individuals were euthanized immediately when displaying symptoms of neurological distress. In selected experiments, mice were monitored using T2-weighted MRI scans on a 9.4T MRI system (Bruker).
Wild animals	No wild animals were used.
Reporting on sex	All experiments were performed using female mice only.
Field-collected samples	No field-collected samples were used in this study.
Ethics oversight	Experiments involving mouse orthotopic xenografts were approved by the Ethical Committee for Animal Research in Lund-Malmö (permit numbers 5.8.18-14006/2019 and 5.8.18-01073/2024) and were carried out according to national care regulations of the Swedish Board of Animal and European Union Animal Rights and Ethics Directives.

Note that full information on the approval of the study protocol must also be provided in the manuscript.

Plants

Seed stocks	n/a
Novel plant genotypes	n/a
Authentication	n/a

ChIP-seq

Data deposition

☒ Confirm that both raw and final processed data have been deposited in a public database such as GEO.

☐ Confirm that you have deposited or provided access to graph files (e.g. BED files) for the called peaks.

Data access links<br>*May remain private before publication.*	The CUT&RUN datasets generated in this study have been deposited in GEO (Gene Expression Omnibus) under the accession number GSE300142 (https://www.ncbi.nlm.nih.gov/geo/query/acc.cgi?&acc=GSE300142).

Files in database submission	GSE300142
Genome browser session (e.g. UCSC)	n/a

Methodology

Replicates	Biological duplicates.
Sequencing depth	Library fragment size distribution was assessed via TapeStation High Sensitivity DNA Analysis assay, and libraries were sequenced as paired-end 150 bp (PE150) on a NovaSeqX Sequencing System (Illumina).
Antibodies	HIF-1α (GTX127309, GeneTex).
Peak calling parameters	Peak calling was performed using SEACR (1.3) with a stringent cutoff of FDR < 0.01.
Data quality	Raw sequencing files (FASTQ) were quality-checked using FastQC.
Software	FastQC (quality control), Trimmomatic v0.39 (adapter trimming), Bowtie2 v2.4.5 (alignment), samtools (duplicate removal), deepTools v3.5.5 (signal normalization and scaling), SEACR v1.3 (peak calling), featureCounts / SubRead v2.1.1 (FRiP calculation), ChIPpeakAnno, ChIPseeker (annotation and comparison), EnsDb.Hsapiens.v86, TxDb.Hsapiens.UCSC.hg38.knownGene (gene annotation), Galaxy / UCSC Genome Browser (visualization).

Flow Cytometry

Plots

Confirm that:

- [x] The axis labels state the marker and fluorochrome used (e.g. CD4-FITC).
- [x] The axis scales are clearly visible. Include numbers along axes only for bottom left plot of group (a 'group' is an analysis of identical markers).
- [ ] All plots are contour plots with outliers or pseudocolor plots.
- [x] A numerical value for number of cells or percentage (with statistics) is provided.

Methodology

Sample preparation	The samples used in this study were derived from cell cultures treated as described in the Methods section. Cells were detached using either 0.5 mM EDTA (for surface antigen analysis) or trypsin (for lipid particle uptake experiments). Further details are provided in the Methods section "Flow cytometry analysis".
Instrument	All analyses were performed with Accuri C6 Flow Cytometer (BD Biosciences).
Software	All flow cytometry data were analyzed by using BD CSampler™ Plus Software v1.0.27.1 (BD Biosciences) and FlowJo (v10).
Cell population abundance	At least 10,000 cells were analyzed per biological replicate. Depending on the experiment, PE, FITC or AlexaFluor488 signal were considered for analysis.
Gating strategy	Cells were gated to exclude dead cells/debris based on FSC-H/SSC-H.

- [ ] Tick this box to confirm that a figure exemplifying the gating strategy is provided in the Supplementary Information.

Magnetic resonance imaging

Experimental design

Design type	Animal brain imaging.
Design specifications	T2 weighted 9.4T imaging
Behavioral performance measures	n/a

Acquisition

Imaging type(s)	Structural.
Field strength	9.4 T
Sequence & imaging parameters	Imaging was performed on a 9.4 T Agilent magnet (Agilent, Santa Clara, USA) equipped with Bruker BioSpec AVIII

Sequence & imaging parameters	electronics operating with ParaVision (PV) 7.0.0 and a BGA 12S HP gradient system (Bruker, Ettlingen, Germany) and a mouse brain cryo coil was used.<br>Scanning sequence, RARE, RARE factor 8; TR, 2200ms; TE, 35ms; FOV, 18x18 mm; Resolution, 53 x 53um2; Matrix size, 340 x 340; Slice thickness, 0,7mm; Number of slices, 8; Number of average, 4.
Area of acquisition	Brain.

Diffusion MRI ☐ Used ☒ Not used

Preprocessing

Preprocessing software	n/a
Normalization	n/a
Normalization template	n/a
Noise and artifact removal	n/a
Volume censoring	n/a

Statistical modeling & inference

Model type and settings	n/a
Effect(s) tested	n/a

Specify type of analysis: ☒ Whole brain ☐ ROI-based ☐ Both

Statistic type for inference<br>(See Eklund et al. 2016)	n/a
Correction	n/a

Models & analysis

n/a	Involved in the study
☒	☐ Functional and/or effective connectivity
☒	☐ Graph analysis
☒	☐ Multivariate modeling or predictive analysis
