# Supplementary material for: Tumour acidosis remodels the glycocalyx to control lipid scavenging and ferroptosis
Source: Nat Cell Biol. 2026 Feb 11;28(3):567–80. doi: 10.1038/s41556-026-01879-y (PMC12992114; doi:10.1038/s41556-026-01879-y)

Fig. 3b

Membranes were either cut or stripped
and reprobed to assess different markers.

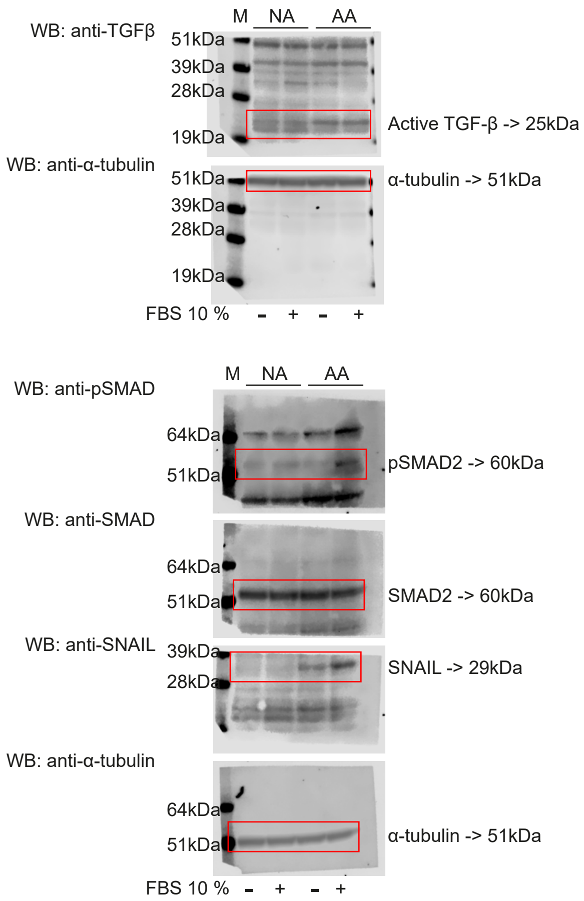

Fig. 3g

Membranes were stripped and
reprobed to assess different markers.

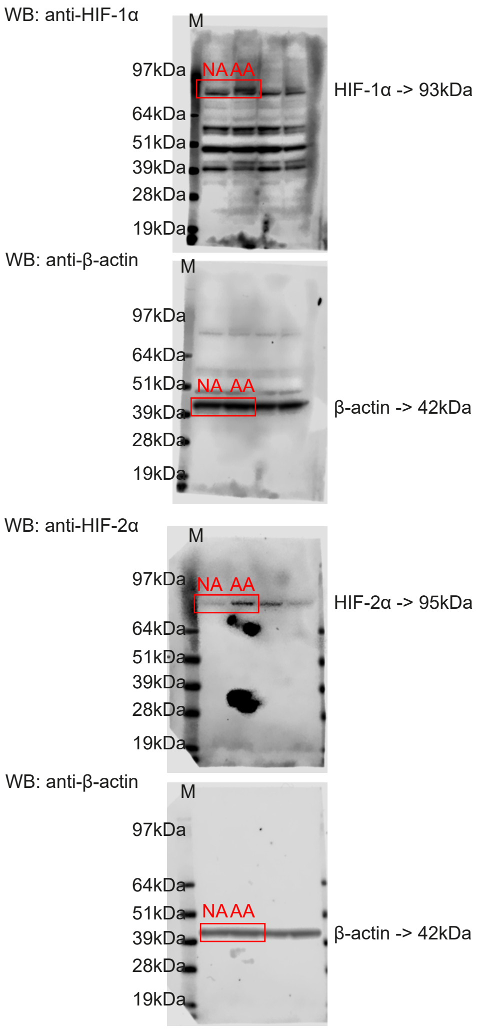

Supplement: Supplementary file 6 — Unprocessed western blots. [file 41556_2026_1879_MOESM6_ESM.pdf]
