# Supplementary figures and images for: Tumour acidosis remodels the glycocalyx to control lipid scavenging and ferroptosis
Source: Nat Cell Biol. 2026 Feb 11;28(3):567–80. doi: 10.1038/s41556-026-01879-y (PMC12992114; doi:10.1038/s41556-026-01879-y)

Fig. 5h

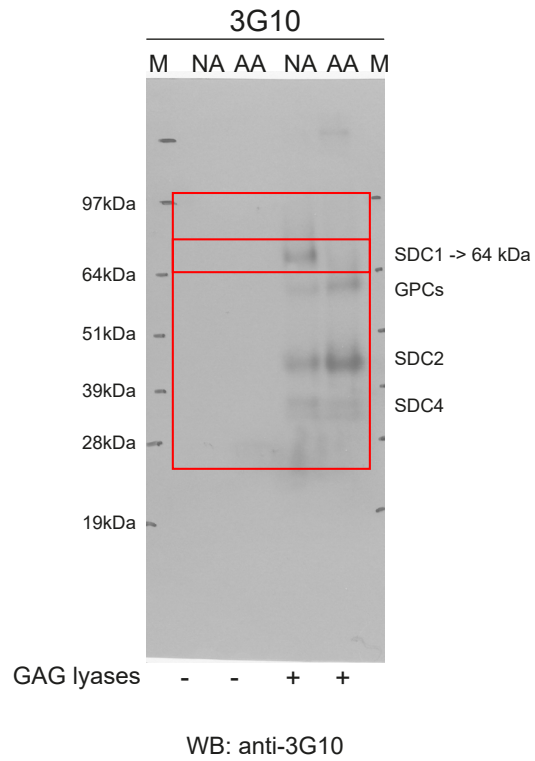

Supplement: Supplementary file 9 — Unprocessed western blots. [file 41556_2026_1879_MOESM9_ESM.pdf]

## Extended data Fig. 6b

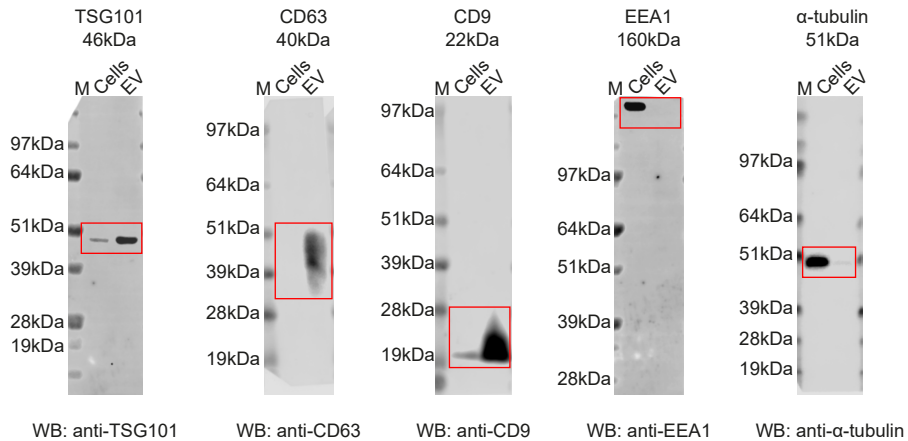

## Extended data Fig. 6j

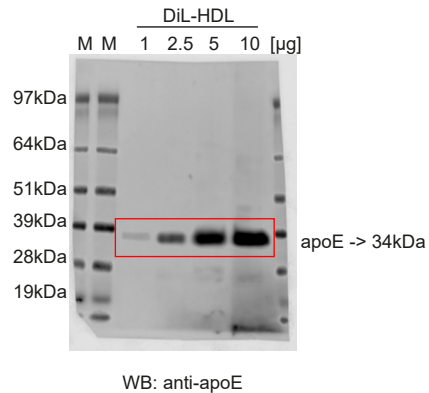

Supplement: Supplementary file 19 — Unprocessed western blots. [file 41556_2026_1879_MOESM19_ESM.pdf]

Extended data Fig. 7g

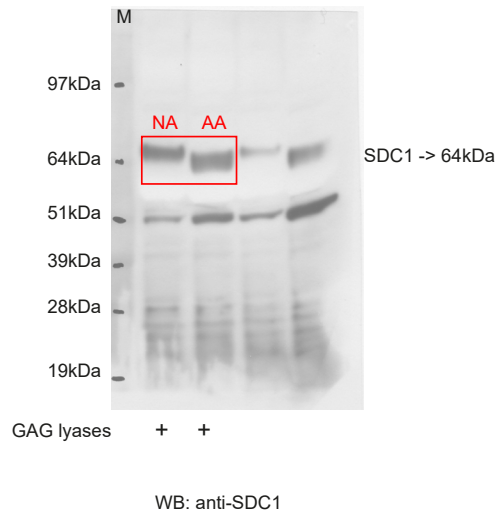

Supplement: Supplementary file 21 — Unprocessed western blots. [file 41556_2026_1879_MOESM21_ESM.pdf]

## Extended data Fig. 9e

Membranes were cut to assess different markers.

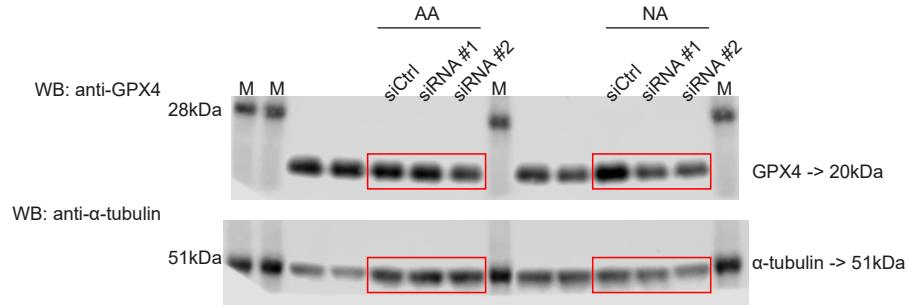

Supplement: Supplementary file 24 — Unprocessed western blots. [file 41556_2026_1879_MOESM24_ESM.pdf]
