# Supplementary material for: Tumour acidosis remodels the glycocalyx to control lipid scavenging and ferroptosis
Source: Nat Cell Biol. 2026 Feb 11;28(3):567–80. doi: 10.1038/s41556-026-01879-y (PMC12992114; doi:10.1038/s41556-026-01879-y)

Extended data Fig. 5b

Membranes were stripped and reprobed to assess different markers.

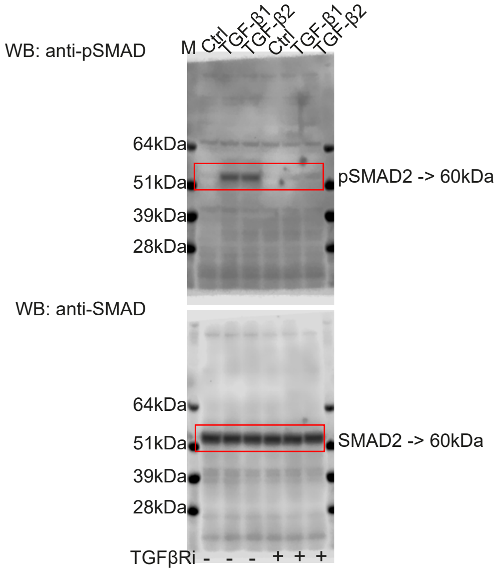

Extended data Fig. 5c

Membranes were cut to assess different markers.

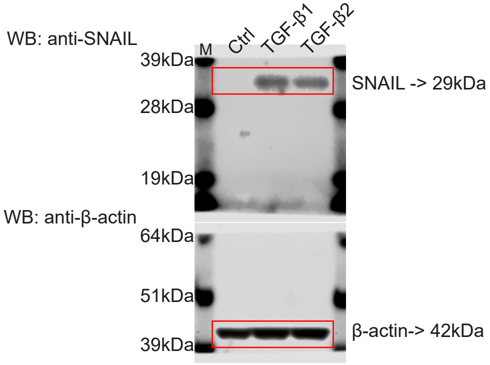

Extended data Fig. 5g

Membranes were cut to assess different markers.

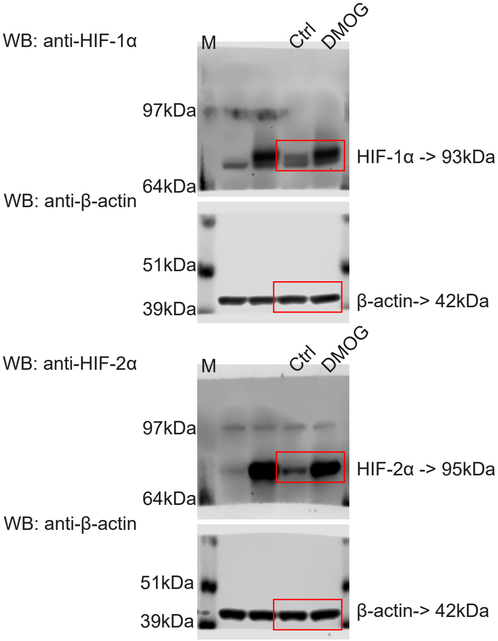

Supplement: Supplementary file 17 — Unprocessed western blots. [file 41556_2026_1879_MOESM17_ESM.pdf]
